# Expression of CD44v6 and integrin-β1 for the prognosis evaluation of pancreatic cancer patients after cryosurgery

**DOI:** 10.1186/1746-1596-8-146

**Published:** 2013-09-02

**Authors:** Gang Zhou, David Chiu, Dajiang Qin, Lizhi Niu, Jinlei Cai, Lihua He, Dongfeng Tan, Kecheng Xu

**Affiliations:** 1 Department of Oncology, The GIBH Affiliated Fuda Hospital, Chinese Academy of Sciences, Guangzhou 510305, China; 2 Department of Oncology, Fuda Cancer Hospital, Guangzhou 510665, China; 3 Department of Pathology, MD Anderson Cancer Center, TX 77030, USA

**Keywords:** PC, CD44v6, Integrin-β1, mRNA, Protein, Cryosurgery

## Abstract

**Background:**

Many previous studies demonstrated that cell adhesion molecules CD44v6 and integrin-β1 had been extensively investigated as potential prognostic markers of various cancers. However, data in PC are scarce.

**Methods:**

We now investigate CD44v6 and integrin-β1 mRNA expression in PBMC by a triplex real-time RT-PCR assay and protein expression in plasma by ELISA. All specimens were collected from 54 PC patients who received the treatment of cryosurgery as well as 20 healthy individuals (control).

**Results:**

The mRNA and protein expression levels of CD44v6 and integrin-β1 in patients were significantly increased compared with control group (*P*<0.05). The high CD44v6 mRNA and protein expression were significantly correlated with clinical stage, tumor differentiation, LNM, liver metastasis and decreased median DFS (*P*<0.05), while the high integrin-β1 mRNA and protein expression were significantly correlated with clinical stage, LNM, liver metastasis and decreased median DFS (*P*<0.05). Clinical stage, LNM, liver metastasis, CD44v6 mRNA and protein expression were the independent predictors of survival in PC patients (*P*<0.05). Moreover, CD44v6 and integrin-β1 mRNA and protein expression levels were significantly decreased in patients in 3 months after cryosurgery (*P*<0.05). No significant difference was found in CD44v6 mRNA and protein expression between patients in 3 months after cryosurgery and control group (*P*>0.05).

**Conclusion:**

CD44v6 and integrin-β1 mRNA and protein expression in blood may serve as biomarkers for the development and metastasis of PC, and as prognostic indicators for PC. They may become useful predictors in assessing outcome of PC patients after cryosurgery.

**Virtual slides:**

The virtual slides for this article can be found here: http://www.diagnosticpathology.diagnomx.eu/vs/4035308681009006.

## Introduction

Pancreatic cancer (PC) is one of the most lethal and aggressive human solid malignancies. The mortality rate of PC ranks in the top 4 in the world and its 5-year survival rate is less than 5% [1]. At present, cancer resection is considered as the main possible solution for the treatment of PC. However, the early diagnosis of PC is extremely difficult and only 10% to 20% patients can be treated with surgery [2]. In addition, the treatment effect is often disappointing due to the postoperative complications [3]. For the other rare type of PC-like pancreatic neuroendocrine tumors, the overall prognosis of them after resection is much better than that of other pancreatic tumors, although they account for less than 3% of PC [4]. Previous study of 3851 cases confirmed that the 5- and 10-year survival rates were 59.3% and 37.7% [5].

It has been confirmed that argon-helium cryosurgery technology is an effective way to treat benign and malignant tumors, especially the tumors which can not be resected by surgery. This technology has been adopted to treat liver cancer, lung cancer and colorectal cancer [6-9]. These studies indicated that in 24 colorectal cancer patients performing cryosurgery, the 1- and 3-year survival rates were respectively 90.8% and 59%. A total of 12 non-small cell lung cancer patients underwent cryosurgery, showing that the 1-year survival rate was 83.3% and no significant complications or adverse reaction occurred during cryosurgery [6]. In contrast, PC cryosurgery treatment is seldom reported [10]. The efficacy and safety of cryosurgery on the treatment of PC require to be proven by new experimental data.

Investigations into the biological mechanisms underlying PC have identified the new and more accurate biological markers and factors of PC, which have been used as important indicators to evaluate the efficacy of therapeutics. Tumor invasion and metastasis originate from the interaction between tumor cells and the extracellular matrix. The key factor is the detachment of tumor cells from the primary tumor and then attaching to the metastasis organs. Cell adhesion molecules CD44 and integrins are a kind of transmembrane glycoprotein on the cell surface. Due to their essential effects in adhesion and signal conduction between tumor cells and the extracellular matrix, they play a key role in tumor invasion and metastasis [11,12]. CD44v6 is a variant of CD44, while integrin-β1 is a major subunit of the integrin family. Recent studies show that CD44v6 and integrin-β1 expression are closely correlated with the progression, metastasis and prognosis of malignant tumors including lung cancer, liver cancer, stomach cancer, breast cancer and colon cancer [13-17]. However, few studies have focused on the role of CD44v6 and integrin-β1 in PC progression and metastasis [18].

In this study, several experiments were performed to better understand the roles of CD44v6 and integrin-β1 gene in the invasion and metastasis of PC. A total of 54 PC patients with different metastatic and invasive abilities and 20 healthy individuals were enrolled in this study. A new triplex real-time RT-PCR assay was successfully developed in the early study to measure the expression levels of CD44v6 and integrin-β1 gene mRNA accurately in the peripheral blood of PC patients. Besides, the levels of CD44v6 and integrin-β1 protein expression in plasma were determined by ELISA assay. The study explored the relationship between CD44v6 and integrin-β1 expression and tumor stage, differentiation, metastasis and survival to determine whether CD44v6 and integrin-β1 can be utilized as new biological markers for the prognostic evaluation of PC. The dynamic change of CD44v6 and integrin-β1 expression in PC patients after cryosurgery was analyzed to identify the potential values of CD44v6 and integrin-β1 in evaluating the outcome of cryosurgery in PC patients.

## Materials and methods

### Patients

From July 2008 and December 2012, 54 PC patients, who underwent cryosurgery at the department of Oncology, Guangzhou Fuda Hospital, were enrolled in this study. The patients included 27 males and 27 females with a median age of 56 years old (33~76 years old). All patients underwent cryosurgery and received no other treatment like chemotherapy, radiation therapy or immunotherapy before and after cryosurgery. Patients were clinically diagnosed by several imaging techniques, including positron emission computed tomography (PET), computed tomography (CT) and magnetic resonance imaging. The diagnosis was histologically confirmed by pancreatic biopsy. All the tissues obtained from the patients were proved to be PC by histopathology. The stage of tumors was assessed according to the American Joint Committee on Cancer (AJCC) guidelines [19]. Pathological characteristics of the tumors collected from 54 patients are listed in Table 1. A group of healthy individuals (control group) randomly selected from hospital staff were asked to participate in the study, including 14 males and 6 females (from 32~65 years old; median age of 44.3 years old). Informed consent was obtained from each patient and healthy individual. Protocols conformed to the ethical guidelines of the 1975 Declaration of Helsinki and were approved by Research Ethics Committee of the Fuda Hospital of the Chinese Academy of Sciences.

**Table 1:** Clinicopathologic characteristics of 54 pancreatic cancer patients

**Characteristics**	**Number of patients (%)**
Sex	
Male	27 (50.0%)
Female	27 (50.0%)
Age	
≤60	24 (44.4%)
>60	30 (55.6%)
Histological diagnosis	
Head PC	17 (31.5%)
Body PC	17 (31.5%)
Tail PC	20 (37.0%)
Clinical stages	
I (T1N0)	8 (14.8%)
II (T2N0, T3N0, T1-2N1)	13 (24.1%)
III (T3N1)	10 (18.5%)
IV	23 (42.6%)
Tumor differentiation	
Poorly	14 (25.9%)
Moderately	19 (35.2%)
Highly	21 (38.9%)
Tumor size (diameter)	
≤4 cm	25 (46.3%)
>4 cm	29 (53.7%)
Lymph node metastasis	
Negative	20 (37.0%)
Positive	34 (63.0%)
Liver metastasis	
Negative	24 (44.4%)
Positive	30 (55.6%)
CA199 (U/ ml)	
≤1000	18 (33.3%)
>1000	36 (66.7%)
KPS	
≤80	25 (46.3%)
>80	29 (53.7%)

Whole peripheral blood samples (5 mL) were collected and transferred into a tube containing EDTA from healthy individuals and 54 patients in 1 day prior to cryosurgery. The blood samples were collected and performed from all patients in 10 days after cryosurgery, in 1 month after cryosurgery, in 3 months after cryosurgery and in 6 months after cryosurgery as described above. Blood samples were used to isolate PBMC and plasma. The mRNA and protein expression levels of CD44v6 and integrin-β1 in PBMC and plasma were measured.

### Treatment and follow-up

The cryosurgery procedure was performed under local anesthesia and under the guidance of ultrasound or CT. Cryoprobe insertion was often carried out via the retroperitoneal approach. Generally, 2 or 3 mm cryoprobes were used. For the tumors greater than 3 cm in size, 2 to 3 probes were used. For liver metastases, simultaneous cryosurgery was performed with additional cryoprobes, which were inserted through the right intercostal space. The cryosurgery procedure was similar to that performed in previous study [6].

Patients underwent cryosurgery as appropriate for their diagnosis and disease evaluation by their medical oncologist according to the guidelines of hospital. The evaluation consists of physical examination, a complete blood count, blood chemical tests, screening serum tumor markers, computed tomographic scan and magnetic resonance imaging according to tumor stage in a 2-month interval. The planned reevaluation for patients with metastatic disease was performed every 1 month. Close follow-up was documented by contacting family practitioners with questionnaires concerning local relapse, distant metastasis and death.

### Isolation of PBMCs and plasma

The whole peripheral blood (5 mL) was diluted with the equal volume of phosphate-buffered saline (PBS, pH=7.3), stratified on a Ficoll-Hypaque gradient (Invitrogen, Shanghai, China) according to the manufacturer’s protocol, and then centrifuged at 2000 rpm for 20 min. The PBMC and plasma fraction were obtained and the mononuclear cells were washed twice with PBS and resuspended in RPMI 1640 containing 10% DMSO. Each PBMC was counted and divided into two parts (1×10^6^ cells/each part). One part containing 1×10^6^ cells was used for the extraction of the total RNA and the other part was stored in liquid nitrogen. Plasma samples were divided into 2 parts and stored in -80°C for use.

### Investigation of mRNA expression levels of CD44v6 and integrin-β1 in PBMC by quantitative real-time RT-PCR assay

A triplex quantitative TaqMan real-time RT-PCR assay was used to determine CD44v6 and integrin-β1 mRNA expression levels in PBMC [20]. For each sample, the expression levels of CD44v6 and integrin-β1 gene were expressed as the number of CD44v6 or integrin-β1 copies per 10^6^*β-actin* copies. Each patient underwent serial measurements before and after cryosurgery and the change of expression levels over time was analyzed.

### Investigation of protein expression levels of CD44v6 and integrin-β1 in plasma

An ELISA kit (Jingtian Biotech, Shanghai, China) was used to measure the protein expression levels of CD44v6 and integrin-β1 in plasma. The ELISA was performed by coating 96-well plates with 2.5 ng/well of capture Ab. Before the subsequent steps, the coated plates were washed twice with PBS containing 0.05% Tween-20 (PBST). All reagents and coated wells used were incubated for 1 hour at room temperature. The standard curve was generated from the serially diluted standard samples provided by the manufacturer. After exposure to the medium, the plates were exposed sequentially to each of the biotin-conjugated secondary antibodies, avidin peroxidase and TMB substrate solution according to the instructions of the ELISA kit. The reaction is terminated by the addition of a sulphuric acid solution and the color change is measured spectrophotometrically at a wavelength of 450 nm by thermo MK3 microplate reader (Thermo Labsystems, Foster, USA). Then the concentrations of CD44v6 and integrin-β1 in the samples are obtained through comparing the O.D. of the samples according to the standard curve constructed with standard samples.

### Statistical analysis

SPSS 17.0 software is adopted for statistical analysis. Log transformations of the CD44v6 and integrin-β1 mRNA expression levels were performed to normalize the frequency. The maximal method of Miller [21] and Siegmund and Halpern [22] was adopted to determine which cutoff value best dichotomized patients into low and high expression groups of CD44v6 or integrin-β1. Student’s *t* test was used to evaluate the differences in CD44v6 and integrin-β1 expression levels between PC patients and control group. Chi-square test was used to assess the relationships between CD44v6 and integrin-β1 expression levels and the clinicopathological factors. DFS and OS were estimated according to the Kaplan-Meier Method and the association of CD44v6 and integrin-β1 expression with DFS and OS was examined by a log-rank test. OS was obtained from the day of surgery to the day of death or the last follow-up. DFS was obtained from the day of surgery to the day of the first documented relapse or death or the last follow-up. Prognostic factors were determined through Cox regression analysis. The nonparametric method was adopted to analyze the differences in CD44v6 and integrin-β1 expression levels among different time points. A p-value less than or equal to 0.05 was considered statistically significant.

## Results

### CD44v6 and integrin-β1 mRNA and protein expression levels

**Figure 1:**
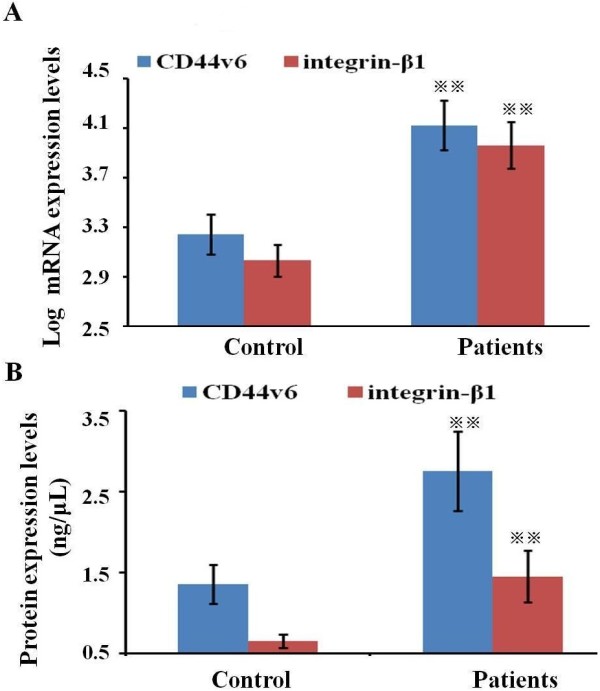
**The comparison of CD44v6 and integrin-β1 expression levels between patients and control. (A)** CD44v6 and integrin-β1 mRNA expression levels in PBMC of patients and control were determined by the triplex quantitative RT-PCR. **(B)** CD44v6 and integrin-β1 protein expression levels in plasma of patients and control were determined by the quantitative ELISA. Notes: Bars indicate standard error and ^※※^ symbols indicate the statistical difference between patients and control (^※※^*p*<0.01).

The median CD44v6 and integrin-β1 mRNA expression levels were 4.12 (range 2.57–5.43) and 3.96 (range 2.12–5.21). According to the cutoff values of CD44v6 and integrin-β1, the low and high CD44v6 mRNA expression levels were respectively found in 32 (59.3%) and 22 (40.7%) patients, while the low and high integrin-β1 mRNA levels were respectively found in 33 (61.1%) and 21 (38.9%) patients. The mRNA levels of CD44v6 and integrin-β1 in patients with PC were significantly higher than those in control group (*P*<0.01) (Figure 1A). The median protein levels of CD44v6 and integrin-β1 in patients were 2.75 (range of 1.34-4.53) and 1.45 (range of 0.69-3.36). According to the cutoff values of CD44v6 and integrin-β1, the low and high CD44v6 protein expression levels were respectively found in 30 (55.6%) and 24 (44.4%) patients, while the low and high integrin-β1 protein levels were respectively found in 27 (50.0%) and 27 (50.0%) patients. The protein expression levels of CD44v6 and integrin-β1 in patients with PC were significantly higher than those in the control group (*P*< 0.01) (Figure 1B).

### Relationship between CD44v6 or integrin-β1 expression levels and clinical pathological features of patients with PC

Statistical analysis showed that the expression levels of CD44v6 mRNA or protein were significantly correlated with clinical stage, tumor differentiation, LNM and liver metastasis (*P*<0.05). No significant correlation was found between CD44v6 mRNA or protein expression and other clinical pathological features of sex, age, tumor location, tumor size, CA199 levels and KPS scores (*P*>0.05). In addition, both integrin-β1 mRNA and protein expressions were correlated with clinical stage, LNM and liver metastasis (*P*<0.05). No significant correlation was observed between integrin-β1 mRNA or protein expressions and other clinical pathological features (*P*>0.05) (Table 2 and Table 3).

**Table 2:** The relationship between CD44v6 and integrin-β1 mRNA expression and clinical pathological features of patients with PC

**Pathological features**	**N**	**CD44v6 mRNA expression**		***P *value**	**Integrin-β1 mRNA expression**		***P *value**
		**High**	**Low**		**High**	**Low**	
Sex				0.238			0.132
Male	27	12 (54.5%)	15 (45.5%)		13 (48.1%)	14 (51.9%)	
Female	27	10 (37.0%)	17 (63.0%)		8 (29.6%)	19 (70.4%)	
Age				0.921			0.810
≤60	24	10 (41.7%)	14 (58.3%)		9 (37.5%)	15 (62.5%)	
>60	30	12 (40.0%)	18 (60.0%)		12 (40.0%)	18 (60.0%)	
Tumor location				0.235			0.241
Head and Body PC	34	12 (35.3%)	22 (64.7%)		14 (41.2%)	20 (58.8%)	
Tail PC	20	10 (50.0%)	10 (50.0%)		7 (35.0%)	13 (65.0%)	
Clinical stages				0.016			0.023
I+II	21	4 (19.0%)	17 (81.0%)		4 (18.2%)	18 (81.8%)	
III+IV	33	18 (54.5%)	15 (45.5%)		17 (53.1%)	15 (46.9%)	
Tumor differentiation				<0.01			0.067
Poorly and Moderately	33	7 (21.2%)	26 (78.8%)		9 (29.0%)	22 (71.0%)	
Highly	21	15 (71.4%)	6 (28.6%)		12 (52.2%)	11 (47.8%)	
Tumor size (diameter)				0.187			0.114
≤4 cm	25	8 (32.0%)	17 (68.0%)		7 (28.0%)	18 (72.0%)	
>4 cm	29	14 (48.3%)	15 (51.7%)		14 (48.3%)	15 (51.7%)	
LNM				0.028			0.034
Negative	20	4 (20.0%)	16 (80.0%)		6 (23.1%)	20 (76.9%)	
Positive	34	18 (52.9%)	16 (47.1%)		15 (53.6%)	13 (46.4%)	
Liver metastasis				0.020			0.028
Negative	24	5 (20.8%)	19 (79.2%)		5 (20.8%)	19 (79.2%)	
Positive	30	17 (56.7%)	13 (43.3%)		16 (53.3%)	14 (46.7%)	
CA199(U/ml)				0.887			0.165
≤1000	18	7 (38.9%)	11 (61.1%)		5 (27.8%)	13 (72.2%)	
>1000	36	15 (41.7%)	21 (58.3%)		16 (44.4%)	20 (55.6%)	
KPS				0.665			0.841
≤80	25	11 (44.0%)	14 (56.0%)		10 (40.0%)	15 (60.0%)	
>80	29	11 (37.9%)	18 (62.1%)		11 (37.9%)	18 (62.1%)	

**Table 3:** The relationship between CD44v6 and integrin-β1 protein expression and clinical pathological features of patients with PC

**Pathological features**	**N**	**CD44v6 protein expression**		***P *value**	**Integrin-β1 protein expression**		***P *value**
		**High**	**Low**		**High**	**Low**	
Sex				0.201			0.269
Male	27	14 (51.9%)	13 (48.1%)		15 (55.6%)	12 (44.4%)	
Female	27	10 (37.0%)	17 (63.0%)		12 (44.4%)	15 (55.6%)	
Age				0.856			0.189
≤60	24	11 (45.8%)	13 (54.2%)		14 (58.3%)	10 (41.7%)	
>60	30	13 (43.3%)	17 (56.7%)		13 (43.3%)	17 (56.7%)	
Tumor location				0.086			0.378
Head and body PC	34	12 (35.3%)	22 (64.7%)		16 (47.1%)	18 (52.9%)	
Tail PC	20	12 (60.0%)	8 (40.0%)		11 (55.0%)	9 (45.0%)	
Clinical stages				0.046			0.019
I+II	21	6 (28.6%)	15 (71.4%)		6 (31.6%)	13 (68.4%)	
III+IV	33	18 (54.5%)	15 (45.5%)		21 (60.0%)	14 (40.0%)	
Tumor differentiation				0.037			0.038
Poorly and moderately	33	10 (30.3%)	23 (69.7%)		12 (36.4%)	21 (63.6%)	
Highly	21	14 (66.7%)	7 (33.3%)		15 (71.4%)	6 (28.6%)	
Tumor size (diameter)				0.112			0.154
≤4 cm	25	8 (32.0%)	18 (68.0%)		11 (44.0%)	14 (56.0%)	
>4 cm	29	16 (55.2%)	12 (44.8%)		16 (55.2%)	13 (44.8%)	
LNM				0.035			0.017
Negative	20	5 (25.0%)	15 (75.0%)		7 (30.4%)	16 (69.6%)	
Positive	34	19 (55.9%)	15 (44.1%)		20 (69.0%)	9 (31.0%)	
Liver metastasis				0.013			0.029
Negative	24	5 (20.8%)	19 (79.2%)		7 (29.2%)	17 (71.8%)	
Positive	30	19 (63.3%)	11 (36.7%)		20 (66.7%)	10 (33.3%)	
CA199(U/ml)				0.187			0.354
≤1000	18	6 (33.3%)	12 (66.7%)		8 (44.4%)	10 (55.6%)	
>1000	36	18 (50.0%)	18 (50.0%)		19 (52.8%)	17 (47.2%)	
KPS				0.233			0.176
≤80	25	13 (52.0%)	12 (48.0%)		15 (60.0%)	10 (40.0%)	
>80	29	11 (37.9%)	18 (62.1%)		12 (41.4%)	17 (58.6%)	

### Relationship between CD44v6 or integrin-β1 expression and survival rate

**Figure 2:**
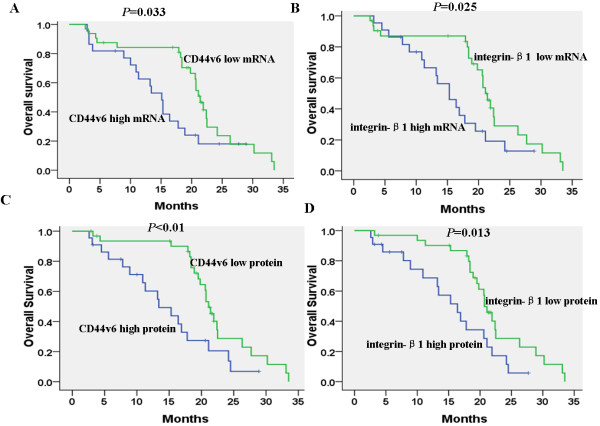
**Kaplan-Meier survival analyses with log-rank statistics according to CD44v6 and integrin-β1 expression in patients. (A-B)** Significant correlation between CD44v6 or integrin-β1 mRNA expression and disease-free survival (DFS) was observed. **(C-D)** Significant correlation was found between CD44v6 or integrin-β1 protein expression and DFS.

The high or low mRNA expression levels of either CD44v6 or integrin-β1 show the significant correlations with median DFS (21.4 months vs. 15.1 months, *P*=0.033; 21.1 months vs. 15.3 months, *P*=0.025). The high or low protein expression levels of either CD44v6 or integrin-β1 also show significant correlations with median DFS (21.1 months vs. 13.4 months, *P*<0.01; 20.9 months vs. 16.4 months, *P*=0.013) (Figure 2).

Univariate analysis revealed that clinical stage, lymph node metastasis, liver metastasis and CD44v6 or integrin-β1 mRNA and protein expression levels were correlated with DFS and OS (*P*<0.05). However, sex, age, tumor location, tumor size, tumor differentiation, CA199 levels and KPS scores were not correlated with DFS and OS (*P*>0.05). In multivariate analysis, clinical stage, lymph node metastasis, liver metastasis and CD44v6 mRNA and protein expression levels were also independent prognostic factors for DFS and OS (*P*<0.05). Integrin-β1 mRNA and protein expression levels were independent prognostic factors for OS (*P*<0.05), but not for DFS (*P>*0.05) (Table 4).

**Table 4:** Univariate and multivariate analyses of recurrence and survival (Cox regression)

**Variables**	**Disease-free survival**		**Overall survival**	
	**HR (95% CI)**	***P *value**	**HR (95% CI)**	***P *value**
**Univariate analysis**				
Sex (Male/Female)	1.5 (0.7–3.5)	0.236	1.7 (0.9–3.5)	0.178
Age (≤60/>60)	0.8 (0.4–2.6)	0.421	1.4 (0.9–3.6)	0.279
Histological diagnosis (Head and Body PC/Tail PC)	1.8 (0.9–3.9)	0.145	1.5 (0.6–3.4)	0.243
Clinical stages (I+II/III+IV)	2.8 (1.4–5.9)	0.018	3.2 (0.9–6.7)	<0.01
Tumor differentiation (Poor and Moderate/High)	1.2 (0.3–3.7)	0.231	1.8 (0.5–4.4)	0.137
Tumor size (≤4 cm/>4 cm )	1.8 (0.8–4.1)	0.165	2.2 (1.2–4.2)	0.089
LNM (Negative/Positive)	3.9 (1.1–8.6)	<0.01	4.5 (1.5–8.7)	<0.01
Liver metastasis (Negative/Positive)	3.5 (1.5–6.4)	<0.01	4.1 (1.6–8.5)	<0.01
CA199(U/ml) (≤1000/ >1000)	2.4 (0.7–4.3)	0.059	2.0 (0.6–4.8)	0.107
KPS (≤80/>80)	1.9 (0.5–4.8)	0.156	2.3 (0.8–4.7)	0.119
CD44v6 mRNA (Low/High)	3.1 (1.2–6.4)	0.017	2.9 (1.4–6.8)	0.033
Integrin-β1 mRNA (Low/High)	2.7 (1.3–6.3)	0.034	3.2 (1.5–6.8)	0.025
CD44v6 protein (Low/High)	3.2 (1.1–7.1)	0.012	3.9 (1.2–7.5)	<0.01
integrin-β1 protein (Low/High)	2.7 (0.8–6.7)	0.045	3.0 (1.1–6.2)	0.013
**Multivariate analysis**				
Clinical stages (I+II/III+IV)	3.5 (0.9–7.5)	<0.01	3.6 (1.1–7.1)	<0.01
LNM (Negative/Positive)	2.8 (0.6–7.2)	0.033	3.2 (1.0–6.9)	0.021
Liver metastasis(Negative/Positive)	3.3 (0.7–6.8)	0.015	3.5 (1.2–7.5)	<0.01
CD44v6 mRNA (Low/High)	2.9 (1.2–6.5)	0.028	2.9 (1.4–6.8)	0.033
Integrin-β1 mRNA (Low/High)	2.4 (0.6–6.7)	0.096	3.2 (1.1–7.2)	0.019
CD44v6 protein (Low/High)	3.3 (1.3–7.8)	0.010	3.7 (1.3–7.5)	<0.01
Integrin-β1 protein (Low/High)	2.5 (0.7–6.8)	0.067	3.2 (1.2–6.7)	0.016

### The change of CD44v6 and integrin-β1 expression of PC patients before and after cryosurgery and control

**Figure 3:**
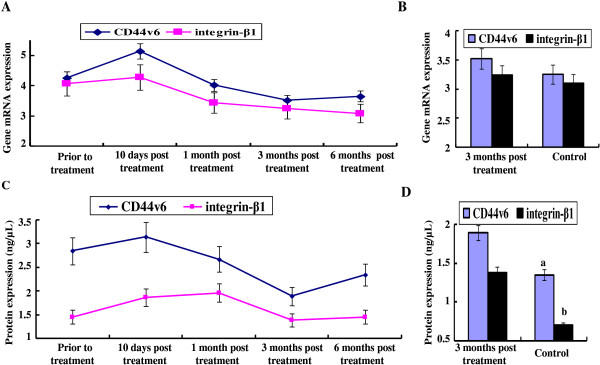
**CD44v6 and integrin-β1 expression levels in patients prior to and after cryosurgery. (A)** The trends of CD44v6 and integrin-β1 mRNA expression levels in patients after cryosurgery were observed. Bars indicate standard error (SE); **(B)** Differences of CD44v6 and integrin-β1 mRNA expression levels between control and patients in 3 months after cryosurgery were analyzed; **(C)** The trends of CD44v6 and integrin-β1 protein expression levels in patients after cryosurgery were observed; **(D)** Differences of CD44v6 and integrin-β1 protein expression levels between control and patients in 3 months after cryosurgery were analyzed (^a^*P*<0.05 and ^b^*P*<0.05 stands for significant difference between two groups).

The dynamic changes of CD44v6 and integrin-β1 mRNA and protein expression levels in patients after cryosurgery treatment were shown in Figure 3. Compared with the patients prior to treatment, CD44v6 levels in patients in 10 days after treatment were significantly increased (*P*<0.05) and integrin-β1 levels in patients in 10 days after treatment showed moderate increase (*P*>0.05). However, both CD44v6 and integrin-β1 levels in patients in 1 month after treatment were significantly decreased compared with patients in 10 days after treatment (*P*<0.05). Their levels in patients in 3 months after treatment were significantly decreased compared with patients before treatment (*P*<0.05) (Figure 3A). Furthermore, no significant difference in CD44v6 and integrin-β1 levels was observed between patients in 3 months after treatment and control group (*P*>0.05, Figure 3B). Both CD44v6 and integrin-β1 protein levels in patients in 3 months after treatment were significantly lower than those in patients before treatment or in patients in 10 days after treatment (*P*<0.05) (Figure 3C). Both CD44v6 and integrin-β1 protein levels in patients in 3 months after cryosurgery were significantly higher than those in the control group (*P*<0.05) (Figure 3D).

## Discussion

Many studies confirmed that the expression abnormalities presented by cell adhesion molecules were closely correlated with the tumor invasion and metastasis, and might be one of the determinants of tumor cells to gain the ability of invasion and distant metastasis. Cell adhesion molecules CD44v6 and integrin-β1 show high expression levels in many tumor cell lines and solid human tumor tissues. In clinical studies, a number of studies addressed the correlation between the two markers (CD44v6 and integrin-β1) and the prognosis of many cancers. However, the roles of CD44v6 and integrin-β1 in predicting the prognosis for advanced PC, especially in predicting prognosis for cryosurgery against PC, were seldom reported. Several studies have reported that blood mRNA, in addition to DNA, are practical markers for clinical use for healthy individuals and for patients suffering from cancer [23,24]. Since tissue specimens must be removed by tumor resection or biopsy, we often can not prepare enough specimens for the detection of biomarker expression, which is essential for the metastasis and prognosis evaluation of many tumors. Therefore, it is important to investigate gene expression in blood samples, instead of gene expression in tumor tissue, and determine the potential roles of related genes as biological markers for the prognosis assessment of PC. Besides, in most of previous studies, animal models and cell lines were adopted as experimental objects to evaluate the role of biomarkers in the prognosis of cancer. However, tumor progression process in animal models and cell lines differed greatly from that of human. Besides, the conventional detection methods used in these studies, such as RT-PCR and immunohistochemistry, may lead to a high false-positive rate in the investigation of clinical samples. Thus, the CD44v6 and integrin-β1 expression levels can not be precisely quantified, resulting in the errors of data analysis in these studies. Hence, the controversy on the roles of CD44v6 and integrin-β1 in tumor metastasis and differentiation are aroused. Currently, the determination of relative gene expression levels through quantitative real-time PCR is considered to be a more sensitive and more quantitative methodology than immunohistochemistry. In this method, an internal reference gene is adopted as a control for DNA quality for each sample assayed. It also acts as internal baseline measurement of the amount of DNA and allows to determining the smaller differences between samples. Thus these results are more precise than immunohistochemistry-measured protein expression. In this study, the mRNA and protein expression levels of CD44v6 and integrin-β1 in 54 PC patients and 20 healthy individuals were determined by the triplex quantitative real-time RT-PCR and quantitative ELISA assay. By constructing standard curves, CD44v6 and integrin-β1 expression levels of samples were exactly tested.

Our study confirmed that both mRNA and protein expression levels of CD44v6 and integrin-β1 in PC patients were significantly up-regulated compared with those in healthy individuals (*P*<0.05), proving the correlation between the two markers (CD44v6 and integrin-β1) and the development of PC, which was consistent with previous results [25]. We also found that the CD44v6 mRNA and protein up-regulated expression was correlated with tumor differentiation, clinical stage, LNM and liver metastasis, implying that CD44v6 could facilitate the proliferation, invasion and metastasis of PC. The rising CD44v6 expression in blood may become one of the important new indicators in predicting early development, metastasis and recurrence of PC. In addition, both integrin-β1 mRNA and protein expression levels were correlated with clinical stage, LNM and liver metastasis (*P*<0.05), indicating that integrin-β1 could increase distant metastasis. The results show that CD44v6 and integrin-β1 may play a more important role in predicting the development and metastasis of PC. Some previous studies have revealed that CD44v6 and integrin-β1 can enhance the adhesion of tumor cells to mesothelial cells and extracellular matrix, promoting the release and activation of proteolytic enzyme in tumor cells, as well as the proliferation of tumor vessels [26]. Tumor cells with abnormal CD44v6 and integrin-β1 expression may be possibly assisted by the “camouflage” from lymphocytes and evade the identification and elimination from the human immune system, thus gaining easier access to lymph nodes and forming the metastasis [27,28].

Previous studies have tested CD44v6 and integrin-β1 expression levels in paraffin-embedded tumor samples in patients with gastric carcinoma and lung cancer and have found a significant correlation between decreasing survival rate and increasing expression levels of CD44v6 and integrin-β1. Our study is the first time to analyze the correlation between CD44v6 or integrin-β1 expression in blood and survival in patients with PC. Our data firstly demonstrated that PC patients with high mRNA and protein expression levels of CD44v6 or integrin-β1 had a significantly reduced survival time than patients with **low** CD44v6 or integrin-β1 expression (*P*<0.05). Univariate and multivariate analyses indicate that CD44v6 mRNA and protein expression can serve as independent prognostic factors for OS and DFS in patients with PC, while integrin-β1 mRNA and protein expression level is prognostic factors for OS, showing that the prediction value of CD44v6 expression on DFS is better than integrin-β1 expression.

Chemotherapy and radiotherapy are the main treatment selection of advanced PC. However, most studies have indicated that median survival time is less than 10 months [29,30]. Therefore, it is necessary to explore a new treatment method of PC. In recent years, argon-helium cryosurgery, as a new tumor ablation technology, has been widely used in the treatment of various solid tumors. However, only a few studies showed the outcome of PC patients after the treatment of cryosurgery [9,31]. In order to analyze the outcome of PC patients after the treatment of cryosurgery, the trends of CD44v6 and integrin-β1 expression after cryosurgery were firstly analyzed. Our results found that mRNA and protein expression levels of CD44v6 or integrin-β1 increased in patients in 10 days after cryosurgery. In cryosurgery, freezing temperature exerts a largely different damaging effect on tumor cells and releases large amounts of intact tumor antigens in the form of necrotic tumor cells, leading to the up-regulated expression of CD44v6 and integrin-β1 in the initial stage after cryosurgery. After then, CD44v6 or integrin-β1 expression levels decreased dramatically in the later stage. Moreover, CD44v6 or integrin-β1 mRNA and protein expression levels in patients in 3 months after treatment were significantly decreased compared with patients before treatment (*P*<0.05). Besides, no obvious difference was found in CD44v6 or integrin-β1 mRNA expression levels between patients in 3 months after treatment and control group (*P*>0.05). In addition, the other parts of PBMC and plasma in 54 PC patients were measured in Stem Cell Research Center, Guangzhou Institutes of Biomedicine and Health, Chinese Academy of Sciences according to the protocol mentioned in the subsection of materials and methods, presenting the same results with our study. The reason is that the initial necrotic tumor cells released into blood are gradually removed by the process of metabolism. Besides, cryosurgery can induce the formation of extracellular and intracellular ice crystals, producing vascular capillary changes and circulatory stagnation. Thus, tumor cells have anti-angiogenesis characteristics and inhibit the differentiation and mature of cell adhesion molecules [32]. At last, CD44v6 and integrin-β1 expression levels in patients in 6 months after treatment showed a moderate increase. This may be related to the recurrence of PC. Since abnormal expression of CD44v6 and integrin-β1 is always related to the poor differentiation, rapid progress, easy metastasis and poor prognosis of PC. Our results demonstrate that cryosurgery may be able to inhibit the development and metastasis of PC. CD44v6 and integrin-β1 expression in blood may become effective biomarkers for evaluating the outcome of cryosurgery in PC patients.

## Abbreviations

PC: Pancreatic cancer; PBMC: Peripheral blood mononuclear cell; ELISA: Enzyme linked immunosorbent assay; LNM: Lymph node metastasis; DFS: Disease-free survival; EDTA: Ethylenediaminetetraacetic acid; OS: Overall survival; DMSO: Dimethyl sulfoxyde; PBS: Phosphate-buffered saline.

## Competing interests

The authors declare that they have no competing interests.

## Authors’ contributions

ZG and XC were the overall principle investigator of the study and were responsible for study design, and interpreted the results. DC participated in the design and performed the statistical analysis. ZG wrote the paper and DC revised it. All authors have read and approved the final manuscript.
